# Correction: Highly sensitive and wearable gas sensors consisting of chemically functionalized graphene oxide assembled on cotton yarn

**DOI:** 10.1039/c8ra90048e

**Published:** 2018-06-07

**Authors:** Min-A. Kang, Seulgi Ji, Seongjun Kim, Chong-Yun Park, Sung Myung, Wooseok Song, Sun Sook Lee, Jongsun Lim, Ki-Seok An

**Affiliations:** Thin Film Materials Research Center, Korea Research Institute of Chemical Technology Daejeon 305-600 Republic of Korea msung@krict.re.kr; Department of Energy Science, Sungkyunkwan University Suwon Gyeonggi-do 440-746 Republic of Korea; Department of Physics, Sungkyunkwan University Suwon Gyeonggi-do 440-746 Republic of Korea

## Abstract

Correction for ‘Highly sensitive and wearable gas sensors consisting of chemically functionalized graphene oxide assembled on cotton yarn’ by Min-A. Kang *et al.*, *RSC Adv.*, 2018, **8**, 11991–11996.

The authors regret that one of the funding bodies listed in the Acknowledgements section of the original article is incorrect. A revised version of the Acknowledgements section, in which ‘NRF-2016M3A7B4900119’ is replaced by ‘NRF-2017M3D9A1073502’, can be found below.

This research was supported by a grant (2011-0031636) from the Center for Advanced Soft Electronics under the Global Frontier Research Program of the Ministry of Science, ICT and Future Planning, Korea. This research was also supported by Nano/Material Technology Development Program through the National Research Foundation of Korea (NRF) funded by the Ministry of Education, Science and Technology (NRF-2017M3D9A1073502).

The Royal Society of Chemistry apologises for these errors and any consequent inconvenience to authors and readers.

## Supplementary Material

